# Energy-Based Wavelet De-Noising of Hydrologic Time Series

**DOI:** 10.1371/journal.pone.0110733

**Published:** 2014-10-31

**Authors:** Yan-Fang Sang, Changming Liu, Zhonggen Wang, Jun Wen, Lunyu Shang

**Affiliations:** 1 Key Laboratory of Water Cycle & Related Land Surface Processes, Institute of Geographic Sciences and Natural Resources Research, Chinese Academy of Sciences, Beijing, China; 2 Key Laboratory of Land Surface Process and Climate Change in Cold and Arid Regions, Chinese Academy of Sciences, Lanzhou, China; Universidad Veracruzana, Mexico

## Abstract

De-noising is a substantial issue in hydrologic time series analysis, but it is a difficult task due to the defect of methods. In this paper an energy-based wavelet de-noising method was proposed. It is to remove noise by comparing energy distribution of series with the background energy distribution, which is established from Monte-Carlo test. Differing from wavelet threshold de-noising (WTD) method with the basis of wavelet coefficient thresholding, the proposed method is based on energy distribution of series. It can distinguish noise from deterministic components in series, and uncertainty of de-noising result can be quantitatively estimated using proper confidence interval, but WTD method cannot do this. Analysis of both synthetic and observed series verified the comparable power of the proposed method and WTD, but de-noising process by the former is more easily operable. The results also indicate the influences of three key factors (wavelet choice, decomposition level choice and noise content) on wavelet de-noising. Wavelet should be carefully chosen when using the proposed method. The suitable decomposition level for wavelet de-noising should correspond to series' deterministic sub-signal which has the smallest temporal scale. If too much noise is included in a series, accurate de-noising result cannot be obtained by the proposed method or WTD, but the series would show pure random but not autocorrelation characters, so de-noising is no longer needed.

## Introduction

Hydrologic process in nature shows complex variability due to the influence of many, often interrelated, physical factors [1] – [2], especially those random and uncertain factors [3] – [4]. Hydrologic time series are the comprehensive reaction of hydrologic process. Therefore, hydrologic time series analysis is always the central topic in stochastic hydrology [5] – [6]. It is to reveal the variability of hydrologic processes, and provide useful information for water activities. In the theory of stochastic hydrology [7], noise is an inevitable component of observed hydrologic data, and it will cause many difficulties in hydrologic series analysis [8] – [9]. Also because of the influence of noise, hydrologic forecasting usually has uncertainty [10] – [11], based on which many hydrologic designs and water resources planning will have risks [12] – [13]. Therefore, de-noising is a required task in hydrologic time series analysis. However, it is a difficult task in practice due to complex composition of hydrologic data and methods' disadvantages.

Presently, there have been a great number of de-noising methods with various theoretical bases. Among them, traditional de-noising methods are mainly based on model simulation or spectral analysis. They are suitable for stationary and linear systems, and depend on the establishment of state space functions [14] – [15], by which accurate de-noising result usually cannot be obtained. Comparatively, wavelet threshold de-noising (WTD) method performs better [16], because it can reveal the localized characteristics of a series both in temporal and frequency domains [17] – [18]. Details of the mathematical fundament of WTD can be found in the literature [19]. Present studies and applications of WTD were reviewed in the literature [9]. However, many key factors influence the efficiency of WTD, including wavelet and decomposition level choice, threshold estimation and coefficient thresholding [20]. De-noising process by WTD is onerous and time-consuming sometimes. Thereby, more effective and easy-to-operate de-noising methods are needed.

By exploiting the advantage of wavelet analysis, the main objective of this paper is to propose an energy-based wavelet de-noising method. A series can be decomposed into a set of sub-signals by discrete wavelet transform (DWT). The variation of energy (*i.e.*, variance) of sub-signal with decomposition level is called “energy distribution” of series in this paper. Energy distributions of hydrologic series and noise were compared by considering uncertainty, based on which an energy-based wavelet de-noising method was proposed. Analyses of many examples indicate the superiority and simplicity of the proposed method compared with WTD. The rest content is organized as follows. Section 2 briefly describes wavelet method. Section 3 estimates energy distribution of noise by operating Monte-Carlo test, based on which Section 4 proposes an energy-based wavelet de-noising method. Section 5 investigates the influences of three key factors on the efficiency of the proposed method. Section 6 discusses the results, and gives several suggestions for improving wavelet de-noising. The study is summarized in the final section.

## Wavelet Transform

Given proper mother wavelet *ψ(t)*, continuous wavelet transform (CWT) of a series *f(t)* can be conducted as [18]:
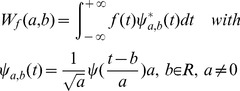
(1)in which ψ^*^(t) is the complex conjugate; *ψ_a,b_(t)* is gained by translating and expanding *ψ(t)*; *a* is temporal scale factor and *b* is time position factor; *W_f_(a,b)* is continuous wavelet coefficient. Observed hydrology series are more often discrete series, and they can be analyzed by DWT:
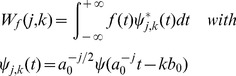
(2)where *a_0_* (*a_0_*>1) and *b_0_* are constants; integer *j* is decomposition level, and *k* is time position factor. The dyadic DWT is used commonly by assigning *a_0_* = 2 and *b_0_* = 1. It consists of log_2_ *n* levels at most given the analyzed series has the length of *n*:



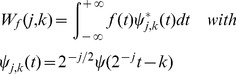
(3)More details of DWT were thoroughly described in [21] – [22]. The wavelet that satisfies regularity condition can be used to reconstruct series *f(t)* [23]:
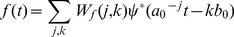
(4)


Wavelet threshold de-noising is based on discrete wavelet result of series. It is to adjust detail wavelet coefficients:
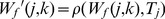
(5)where *ρ()* is the thresholding rule, such as hard-, soft- and mid-thresholding rule, *T_j_* is the threshold under level *j*, and *W_f_'(j,k)* is the adjusted value of *W_f_(j,k)*. De-noised series can be reconstructed by substituting *W_f_' (j,k)* for *W_f_(j,k)* in Eq. (4), and the residue is noise [19]. Wavelet threshold de-noising is influenced by four key factors [24]. The first two are wavelet and decomposition level choice, both determine the accuracy of DWT result in Eq. (2). The other two are threshold estimation and coefficient thresholding, both determine the accuracy of adjusted wavelet coefficients result in Eq. (5).

## Energy Distribution of Noise

In order to establish a reliable basis for wavelet de-noising, Monte-Carlo test is operated to estimate energy distribution of noise. Because probability distribution of noise in hydrologic data is generally unknown, the types of noise following Gauss, 2-parameter lognormal and Pearson-III distributions, G, LN2 and P for short, are analyzed for comparison. The following steps are used for Monte-Carlo test:

1. Generate noise data with the length of *n*, and the biggest decomposition level equals *log*_2_ *n*;
2. Choose proper wavelet, apply dyadic DWT to the noise data using Eq. (3), and obtain detail wavelet coefficients under each decomposition level *j*;
3. Reconstruct sub-signal *f_j_(t)* of noise data under each level *j* by Eq. (4), and calculate its energy (*i.e.*, variance) *E(j)*: 
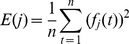
(6)
4. Repeat the steps 1–3 to obtain stable sampling result of Monte-Carlo test. Calculate arithmetic mean of energy of noise's sub-signal under each level *j*, and estimate proper confidence interval (*e.g.*, 95%). The result is taken as energy distribution of noise.

**Figure 1:**
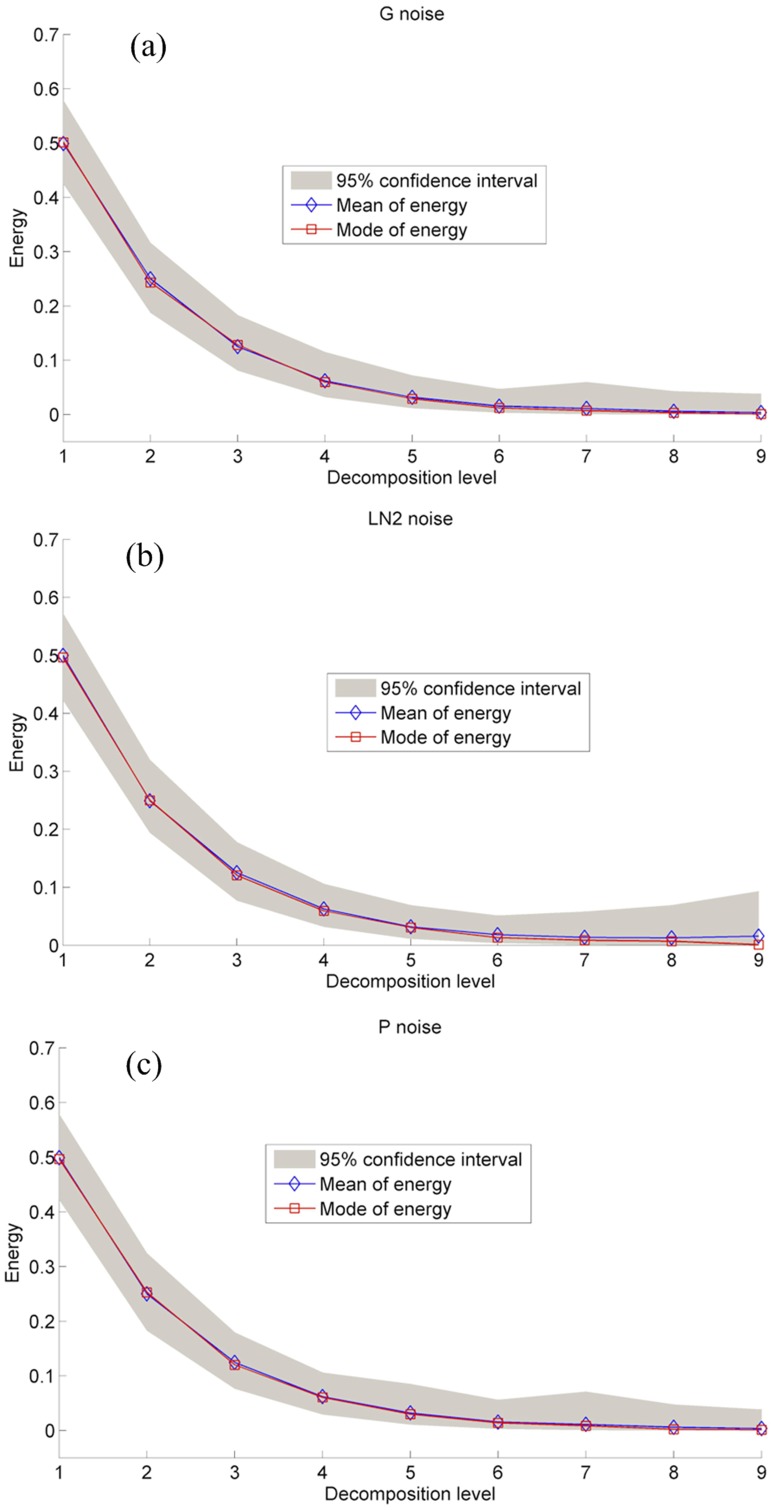
Energy distributions of the gauss (G) (a), 2-parameter lognormal (LN2) (b), and Pearson-III (P) (c) distributed noise with 95% confidence interval.

The above steps are applied to three noise types, and the results are presented in Fig. 1. According to the study in the literature [25], “db5” wavelet is used to analyze these noise data with the same length of 1000. The sampling number of Monte-Carlo test is 50,000 to obtain stable sampling result. The results indicate that arithmetic mean of energy of noise's sub-signal exponentially decreases with level, with the base of 2, and it is due to the grid of dyadic DWT [26] – [27]. Uncertainty can be estimated by determining 95% confidence interval. Arithmetic mean and mode values of energy of sub-signal under each level are the same, so the upper and lower limits of 95% confidence interval are symmetrical to arithmetic mean value. Consequently, energy distributions of three noise types (G, LN2 and P) are similar to each other. They strictly follow the exponentially decreasing rule with the base of 2. Therefore, it is thought that the shape of energy distribution of noise has no relation with noise type.

## Energy-Based Wavelet De-Noising Method Proposed

According to the theory of stochastic hydrology, hydrologic series data is basically composed of deterministic components and noise [7]. The former, mainly including periodicities and trend, are generated by certain physical deterministic mechanisms. Noise, whose existence contaminates true hydrologic data, is generated by many random and uncertain factors. When applying DWT to hydrologic data, energy distributions of the two components are different [28]. Energies of deterministic components concentrate on several decomposition levels, but energy of noise scatters in the whole temporal scales and rapidly decays with level, as displayed in Fig. 1.

Based on their difference of energy distribution, we use the energy distribution of noise to establish a reliable background energy distribution (as defined afterward), and de-noising can be done by comparing energy distribution of series with the background energy distribution. It is the basic idea of the wavelet de-noising method proposed here. Furthermore, we used the confidence interval, which can be estimated from the sampling result of Monte-Carlo test, to evaluate uncertainty in the comparison process.

De-noising steps by the proposed method are explained as follows:

1. Choose proper wavelet, calculate the biggest decomposition level, and apply dyadic DWT to the analyzed series *X* using Eq. (3);
2. Determine the energy distribution of series *X* using Eq. (6);
3. Use proper probability density function to generate noise data *N*, whose length is the same as that of series *X*, then determine the energy distribution of noise data, and estimate proper confidence interval by operating Monte-Carlo test;
4. Set the sub-signal of series *X* under the first level as noise, and use its energy to adjust the energy distribution of noise data and estimated confidence interval. The result is defined as “background energy distribution” in this paper;
5. Compare the energy distribution of series *X* with the background energy distribution. Two cases would be encountered: (a) energy of sub-signal under certain level oversteps the confidence interval. The sub-signal should be a deterministic component; (b) energy of sub-signal under certain level falls within the confidence interval and is close to that of noise. In this case, the sub-signal more likely is composed of noise and should be removed from series *X*;
6. Add up all those deterministic components and obtain the de-noised series, and take the residue as noise.

**Figure 2:**
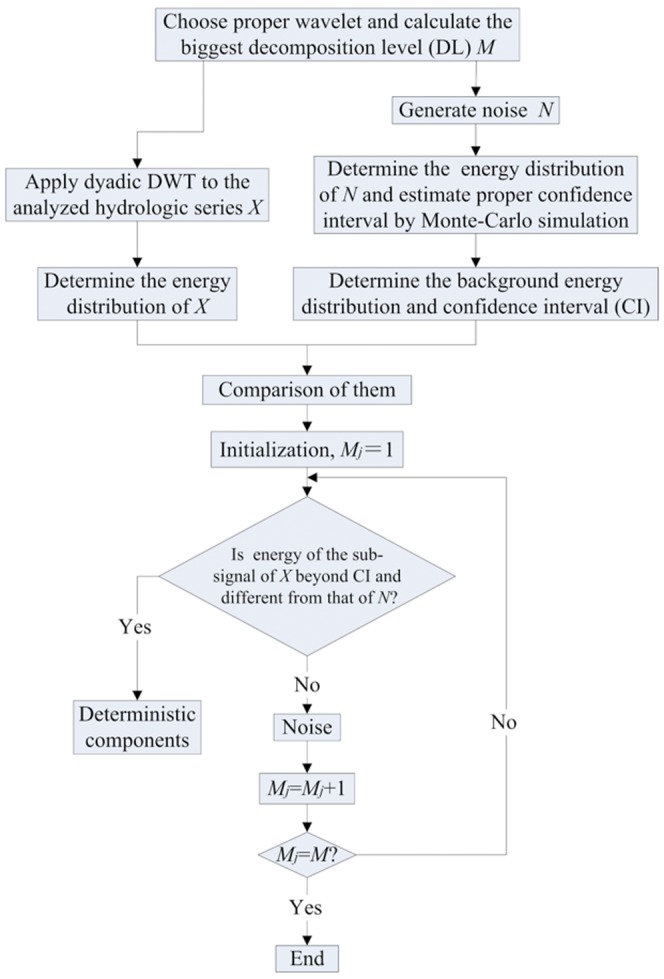
De-noising process of hydrologic time series by the energy-based wavelet de-noising method proposed.

The above de-noising steps by the proposed method are also depicted in Fig. 2.

## Case Study

Both synthetic and observed series are used to verify the performance of the energy-based wavelet de-noising method proposed, and further to investigate the influences of three key factors on wavelet de-noising, including wavelet choice, decomposition level choice and noise content.

Considering that the superiority of WTD method compared with other conventional de-noising methods has been expounded in the literature [9], the latter are omitted here for brevity reason. The energy-based wavelet de-noising method is just compared with WTD. Three indexes, namely SNR (signal-to-noise ratio), MSE (mean square error) and R_xy_ (zero-order cross-correlation coefficient) in Eq. (7), are used to evaluate the de-noising results:
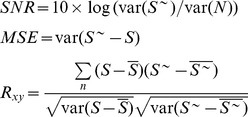
(7)in which 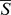 and 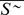 are the mean of true series *S* and de-noised series *S^∼^*, respectively, *N* is the removed noise; *var()* means calculating variance. SNR value reflects noise content of a series. The difference between calculated and true SNR values can reflect the accuracy of de-noising result; MSE value can reflect the similar degree of two series at statistical view, and R_xy_ value can reflect the similar degree of two series at correlation view. Smaller difference between calculated and true SNR, smaller MSE value and bigger R_xy_ value indicate more similarity of two series. Since energy distributions of various noise types are just the same, the Gauss distributed noise is used here, and 95% confidence interval is estimated for uncertainty evaluation.

### Synthetic series analysis

Eight synthetic series in two types are analyzed (Table 1). Four series in type-I have the same length of 1500, and include the same damped period. Four series in type-II have the same length of 500, and include two same true periods of 50 and 200, and the same exponentially upward trend with the base of 1.005. These series include different noise content, and have different true SNR value. All series are analyzed by the proposed method and WTD using various wavelets and decomposition levels. De-noising results are discussed from four perspectives.

**Table 1:** Data used in this paper*.

Type		Data	Length	True components
Synthetic series	Type-I	SS1	1500	The same damped period
		SS12	1500	
		SS13	1500	
		SS14	1500	
	Type-II	SS2	500	The same two periods of 50 and 200
		SS22		
			500	The same exponentially upward trend
		SS23	500	
		SS24	500	
Observed series	Daily temperature (1980–2001)	RS1	8036	Annual period
	Monthly runoff (1972–2001)	RS2	360	12 months

*: Four series in Type-I have no trend, but four series in Type-II have the same exponentially upward trend with the base of 1.005. These synthetic series include different contents of normally distributed noise (i.e., different true SNR values). The RS1 data were gained from the China Meteorological Data Sharing Service System (http://cdc.cma.gov.cn/). The RS2 data were gained from the Center for Water Resources Research, Chinese Academy of Sciences (http://www.cwrr.cn/).

#### Results by different wavelets

**Figure 3:**
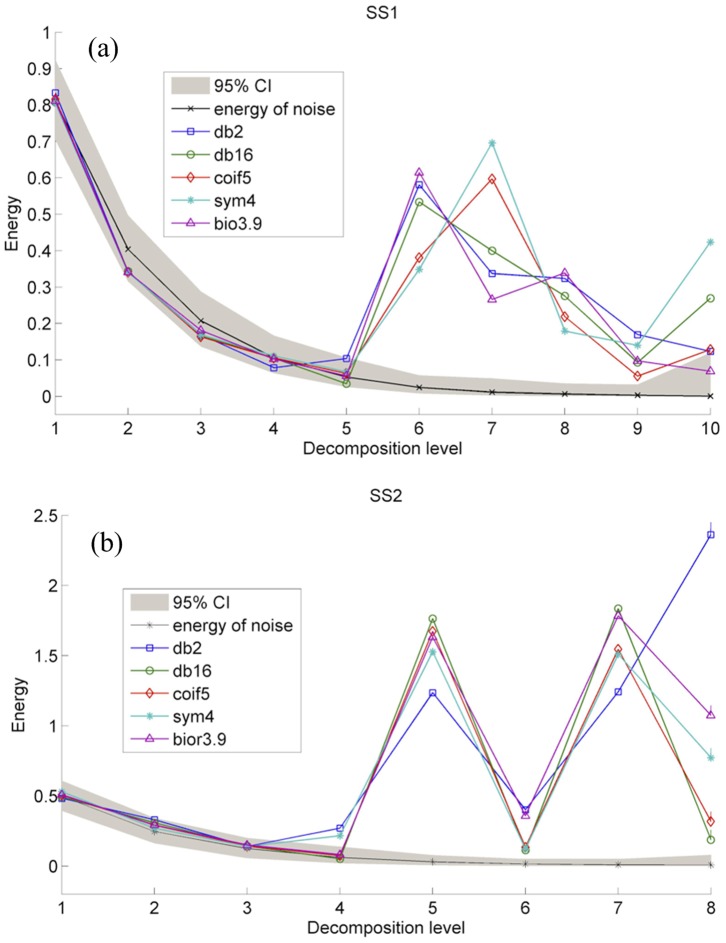
Energy distributions of SS1 (a) and SS2 (b) series obtained by different wavelets.

The decomposition level 10 (*log*_2_1500) is used, and five wavelets (“db2”, “db16”, “sym5”, “coif4” and “bior3.9”) are used to remove noise in SS1 series by the proposed method. Energy distributions of SS1 series obtained by five wavelets are depicted in Fig. 3(a), in which sub-signals under ten levels (Ls) are reconstructed by detail wavelet coefficients. When applying different wavelets to SS1 series, energies of those sub-signals after L6 overstep 95% confidence interval, although their values vary with the wavelet used. Therefore, these sub-signals are regarded as deterministic components, and their sum is the de-noised SS1 series. The same analyses are conducted to SS2 series using the decomposition level 8 (*log*_2_500) and five wavelets. As shown in Fig. 3(b), sub-signals of SS2 series after L5 obtained by any wavelet have the energies overstepping 95% confidence interval, so they are regarded as deterministic components, and their sum is the de-noised SS2 series.

The de-noised SS1 and SS2 series are compared with true series (Table 2). It clearly indicates that de-noising results of each series vary with the wavelet used. Comparatively, de-noising result of SS1 series by “bior3.9” is the best, because the calculated SNR value −0.097 is very close to the true SNR value −0.096. Although de-noising results of SS1 series by “db16” and “bior3.9” wavelets are similar to each other, the calculated SNR value −0.068 by the latter has big difference with the true SNR value −0.096. Therefore, “bior3.9” is chosen as the best wavelet for de-noising SS1 series. For SS2 series, “db16” is chosen as the most suitable wavelet, by which the calculated SNR value 1.123 is very close to the true SNR value 1.116, the MSE value is as little as 0.082, and the R_xy_ value is as big as 0.997.

**Table 2:** Evaluation of the de-noising results of SS1 and SS2 series obtained by different wavelets*.

Series	Index**	Wavelet used				
		db2	db16	sym4	coif5	bior3.9
	SNR (−0.096)	−0.097	−0.068	−0.071	−0.099	**−0.097**
SS1	MSE	0.286	0.272	0.127	0.238	**0.161**
	R_xy_	0.927	0.935	0.964	0.938	**0.959**
	SNR (1.116)	1.035	**1.130**	1.108	1.128	1.129
SS2	MSE	0.353	**0.085**	0.134	0.093	0.109
	R_xy_	0.987	**0.997**	0.995	0.996	0.996

*: In Table 2–3, the true SNR values of SS1 and SS2 series are −0.096 and 1.116, respectively.

**: In all Tables, “*SNR*” means signal-to-noise ratio, “*MSE*” means mean square error, and “*R_xy_*” means the cross-correlation coefficient between series *x* and *y*.

#### Results by different decomposition levels

The “bior3.9” and “db16” wavelets are chosen, and different decomposition levels are used for de-noising SS1 and SS2 series (Table 3). Along with the increase of decomposition level, more noise in original series is removed, and de-noising results are gradually close to true results. For SS1 series, when the decomposition level equals 5 or bigger values, its de-noising results do not change. For SS2 series, its de-noising results obtained by level 4 or bigger levels are also the same. To sum up, the suitable decomposition level for de-noising does not equal the theoretically biggest level of *log*_2_ *n*, but just is the one which can identify series' deterministic sub-signal under the smallest temporal scale.

**Table 3:** Evaluation of the de-noising results of SS1 and SS2 series obtained by different decomposition levels.

Series	Index	Decomposition level used								
		1	2	3	4	5	6	7	8	9
	SNR	0.384	0.151	0.041	−0.020	**−0.097**	−0.097	−0.097	−0.097	−0.097
SS1	MSE	1.054	0.549	0.290	0.241	**0.161**	0.161	0.161	0.161	0.161
	R_xy_	0.802	0.881	0.932	0.935	**0.959**	0.959	0.959	0.959	0.959
	SNR	1.451	1.232	1.156	**1.130**	1.130	1.130	1.130	1.130	
SS2	MSE	0.551	0.242	0.102	**0.085**	0.085	0.085	0.085	0.085	
	R_xy_	0.980	0.991	0.996	**0.997**	0.997	0.997	0.997	0.997	

#### De-noising results of various series

According to the above result, the “bior3.9” wavelet and decomposition level 5 are used for de-noising type-I series, and the “db16” wavelet and decomposition level 4 are used for de-noising type-II series. De-noising results of all series by the proposed method are shown in Table 4. Along with noise content increase, true signals are gradually submerged by noise, so it becomes more difficult to accurately remove noise. Taking the type-I series for example, when just a little noise included, the SS1 series with the true SNR value −0.096 can be de-noised accurately; when the SNR value decreases to −1.770, de-noising result of SS12 series becomes much bad, the MSE value is as big as 3.586, and the R_xy_ value is reduced to 0.441. When more noise is included, the SNR values of SS13 and SS14 series are smaller than −3.000, their MSE values are bigger than 130, and their R_xy_ values are smaller than 0.300, so accurate de-noising results of SS13 and SS14 series cannot be obtained by the proposed method. The same finding can be obtained from de-noising results of type-II series.

**Table 4:** Evaluation of the de-noising results of synthetic series in two types by the energy-based wavelet de-noising method proposed.

Index*	Type-I				Type-II			
	SS1	SS12	SS13	SS14	SS2	SS22	SS23	SS24
R_1_	0.416	0.027	−0.007	−0.041	0.924	0.137	0.110	−0.005
True SNR	−0.096	−1.770	−3.132	−3.742	1.116	−0.891	−2.235	−4.248
Calculated SNR	−0.097	−1.391	−1.241	−1.395	1.130	−0.856	−1.261	−1.851
MSE	0.161	3.586	130.44	375.29	0.085	4.097	53.508	330.6
R_xy_	0.959	0.441	0.179	0.284	0.997	0.857	0.299	0.170

*: “*R_1_*” means lag-1 autocorrelation coefficient.

#### Results by the proposed method and WTD

De-noising results of eight series by both the proposed method and WTD are presented in Table 5. Because SS14 and SS24 series are contaminated by noise severely, they are not considered here. On the whole, de-noising results of all series by the two methods are similar to each other. For SS1 series example, the SNR, MSE and R_xy_ values obtained by the proposed method are −0.097, 0.161 and 0.959, and those obtained by WTD are similar as −0.086, 0.147 and 0.972. Therefore, it is thought that the energy-based wavelet de-noising method has comparable power as WTD in de-noising of series.

**Table 5:** De-noising results of synthetic series by the energy-based wavelet de-noising method proposed and the wavelet threshold de-noising (WTD) method.

Series	Method used*	SNR		MSE	R_xy_
		true value	Calculated value		
SS1	WTD	−0.096	−0.086	0.147	0.972
	New method	−0.096	−0.097	0.161	0.959
SS12	WTD	−1.770	−1.259	4.853	0.386
	New method	−1.770	−1.391	3.586	0.441
SS13	WTD	−3.132	−1.135	159.81	0.156
	New method	−3.132	−1.241	130.44	0.179
SS2	WTD	1.116	1.131	0.084	0.997
	New method	1.116	1.130	0.085	0.997
SS22	WTD	−0.891	−0.694	5.016	0.871
	New method	−0.891	−0.856	4.097	0.857
SS23	WTD	−2.235	−1.194	131.06	0.261
	New method	−2.235	−1.261	53.508	0.299

*: “New method” is the energy-based wavelet de-noising method proposed.

Comparatively, de-noising process by the proposed method can be conducted following the steps in Fig. 2, and it need not handle wavelet coefficients, so is more practical and easier to be operated than WTD. Moreover, uncertainty of de-noising result can also be estimated by the proposed method using 95% confidence interval, but WTD method cannot do this.

### Observed series analysis

Two observed hydrologic series (RS1 and RS2) are analyzed to further verify the performance of the proposed method. RS1 series presents 22-year (1980–2001) daily average temperature data measured at the Beijing weather station, and it has a dominant annual period due to the annual variability of climatic process. RS2 series presents 30-year (1972–2001) monthly average runoff discharge data measured at the Bengbu hydrologic station in the Huai River in East China, and it has a dominant period of 12 months also due to the annual variability of hydrologic process. Lag-1 autocorrelation coefficients of two series are 0.981 and 0.647 respectively, indicating good autocorrelation. Therefore, the two series are not severely contaminated by noise, and their deterministic components can be identified.

**Figure 4:**
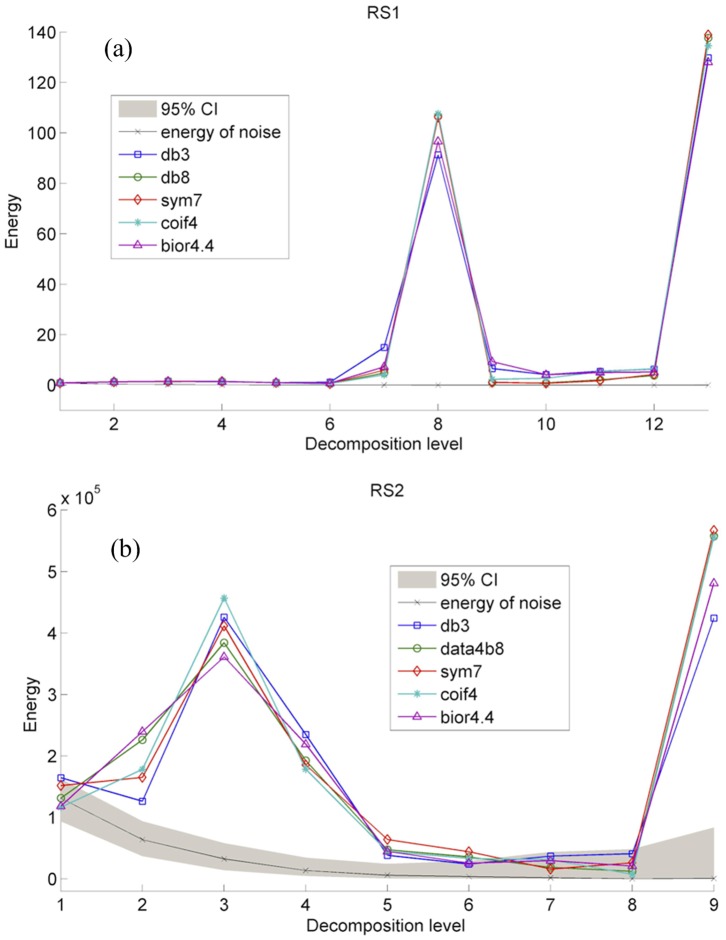
Energy distributions of RS1 (a) and RS2 (b) series obtained by different wavelets.

**Figure 5:**
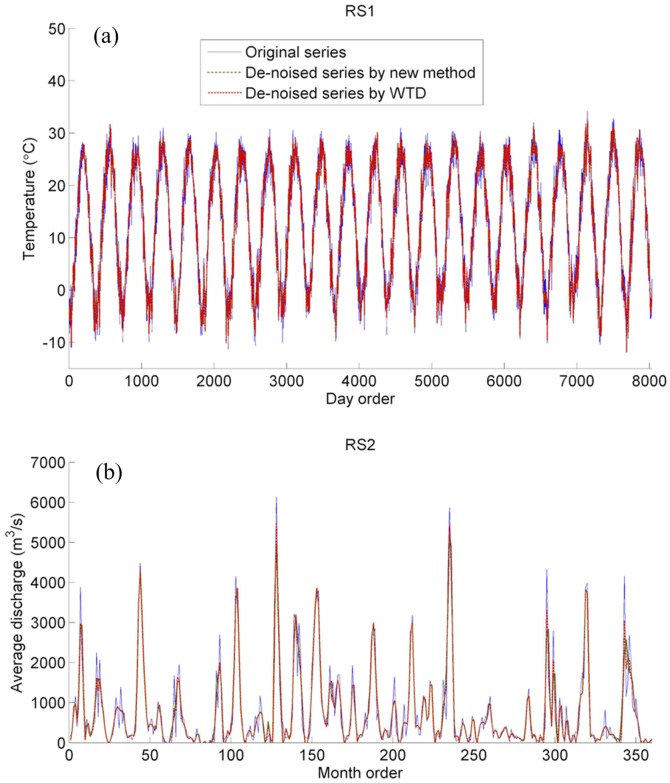
De-noising results of RS1 (a) and RS2 (b) series by the energy-based wavelet de-noising method (the new method) and the wavelet threshold de-noising (WTD) method.

The two observed series are analyzed by the proposed method using five wavelets (“db3”, “db8”, “sym7”, “coif4”, and “bior4.4”). Results in Fig. 4 clearly indicate that energy distributions of series vary with the wavelets used. Energies of RS1 series' sub-signals under L3, L4, L5, L7, L8, L9, L10, L11, and L12 obtained by any of the five wavelets overstep 95% confidence interval, so their sum is the de-noised RS1 series. The sub-signals of RS2 series under L2, L3, L4, L5, L6 and L9 have the energies overstepping 95% confidence interval, and their sum is the de-noised RS2 series. Results in Table 6 indicate that de-noising results of RS1 series by five wavelets are similar, but de-noising results of RS2 series by five wavelets show big difference. It is due to more complex variation of RS2 series as shown in Fig. 5. As a result, the “db8” wavelet is chosen for de-noising of RS1 and RS2 series.

**Table 6:** Evaluation of the de-noising results of RS1 and RS2 series obtained by different wavelets.

Series	Index*	Wavelet used				
		db3	db8	sym7	coif4	bior4.4
	SNR	1.742	**1.742**	1.749	1.751	1.740
RS1	MSE	2.168	**2.167**	2.133	2.124	2.177
	R_xy_	0.991	**0.991**	0.991	0.991	0.991
	SNR	0.732	**0.844**	0.776	0.906	0.900
RS2	MSE (10^5^)	1.651	**1.318**	1.523	1.169	1.188
	R_xy_	0.913	**0.934**	0.922	0.942	0.941

*: In Table 6–7, *MSE* and *R_xy_* are used to compare original series and the de-noised series.

De-noising results of two observed series using different decomposition levels are presented in Table 7. De-noising results of RS1 series using decomposition level 2 or bigger levels are the same. De-noising results of RS2 series using any decomposition level are the same. Because the decomposition levels of 2 and 1 just correspond to the sub-signals which reflect the two observed series' dominant characteristics (*i.e.*, dominant annual period of 12 months), it holds that the de-noising results are reasonable.

**Table 7:** Evaluation of the de-noising results of RS1 and RS2 series obtained by different decomposition levels.

Series	Index	Decomposition level used											
		1	2	3	4	5	6	7	8	9	10	11	12
	SNR	2.171	**1.742**	1.742	1.742	1.742	1.742	1.742	1.742	1.742	1.742	1.742	1.742
RS1	MSE	0.815	**2.167**	2.167	2.167	2.167	2.167	2.167	2.167	2.167	2.167	2.167	2.167
	R_xy_	0.997	**0.991**	0.991	0.991	0.991	0.991	0.991	0.991	0.991	0.991	0.991	0.991
	SNR	**0.844**	0.844	0.844	0.844	0.844	0.844	0.844	0.844	0.844			
RS2	MSE (10^5^)	**1.318**	1.318	1.318	1.318	1.318	1.318	1.318	1.318	1.318			
	R_xy_	**0.934**	0.934	0.934	0.934	0.934	0.934	0.934	0.934	0.934			

The two observed series are also de-noised by WTD (Fig. 5). The calculated SNR values of RS1 and RS2 series obtained by the proposed method are 1.742 and 0.844, and those by WTD are similar as 1.763 and 1.045, being similar with each other. Moreover, de-noised series also have no big difference with original series because of just a little noise included, further indicating the comparable power of the proposed method and WTD.

## Results Discussion

De-noising is an important but difficult task in hydrologic series analysis. By exploiting the advantage of wavelet analysis, an energy-based wavelet de-noising method was proposed in the paper. The influences of three key factors on its efficiency were discussed using various examples. By discussing all results, the following understandings can be gained:

1. Influence of wavelet. Wavelet choice is the foremost task in wavelet analysis [29] – [30], also including wavelet de-noising. When using the energy-based wavelet de-noising method to analyze series, energy distribution and de-noising result of series would vary with the wavelet used. For those synthetic series used in this paper, the most suitable wavelets can be chosen based on de-noising result. However, deterministic components in observed hydrologic data are unknown, so proper wavelet cannot be chosen easily. Here, the criteria proposed in the literature [9] were recommended to choose proper wavelet. The criteria can take the composition and statistical characters of series into account, so they are reliable and useful.
2. Influence of decomposition level. Decomposition level choice is a key factor in discrete wavelet analysis, so it influences wavelet de-noising. Analyses of all series indicate that the most appropriate decomposition level for wavelet de-noising is the one which can identify series' deterministic sub-signal under the smallest scale. If using bigger levels, de-noising results are just the same, but de-noising process would be more time-consuming. Furthermore, the proper decomposition level for energy-based wavelet de-noising is just determined by the composition of the analyzed series, so the chosen decomposition level is reliable, by which de-noising result can be more reasonable.
3. Influence of noise content. Noise type has no influence on the shape of background energy distribution, but noise content in a series determines the magnitude of background energy distribution and confidence interval. Generally, when a series includes too much noise, its deterministic components would be submerged by noise, and energy values of all sub-signals would fall within the estimated confidence interval, so no deterministic component could be identified by the proposed method or WTD. However, for those series which are contaminated severely by noise, they usually show pure random but not good autocorrelation; for instance, the lag-1 autocorrelation coefficient (R_1_) of SS13 and SS14 series is as small as −0.007 and −0.041. Therefore, it need not identify deterministic components in these cases; we can regard them as random series, and use proper statistical methods to describe their statistical characters.
4. Effectiveness of the proposed method. The WTD method used commonly is based on wavelet coefficient thresholding, so its effectiveness is influenced not only by wavelet and decomposition level choice, but also by threshold estimation and coefficient thresholding [19]. Differing from the WTD method, the wavelet de-noising method proposed is based on energy distribution of series, so it has more reliable physical basis. By using it deterministic component of certain series under each level can be identified and separated, and uncertainty can also be estimated. Furthermore, de-noising process by the proposed method can be more easily operable following the steps in Fig. 2.

## Conclusions

In this paper, Monte-Carlo test was operated to estimate energy distribution of noise, and a reliable background energy distribution was established. By comparing energy distribution of series with the background energy distribution, an energy-based wavelet de-noising method was proposed. Its effectiveness and applicability were verified by discussing the influences of three key factors. Results of both synthetic and observed series indicate comparable power of the proposed method as WTD. However, wavelet choice and decomposition level choice should be carefully considered when using the proposed method, and effective methods should be further studied to solve the problem. Those suggestions given above can be used to handle the problem. In addition, we can firstly evaluate autocorrelation of a series. If showing good autocorrelation, the series can be de-noised by the proposed method or other de-noising methods. However, if showing pure random characters, the series can be regarded as a random series, but need not to be de-noised. Further studies using more observed data from other study areas may be required to strengthen the applicability of the energy-based wavelet de-noising method proposed.
